# The 150 most important questions in cancer research and clinical oncology series: questions 15–24: Edited by *Chinese Journal of Cancer*

**DOI:** 10.1186/s40880-017-0205-8

**Published:** 2017-04-05

**Authors:** Chinese Journal of Cancer

**Affiliations:** Editorial Office of Chinese Journal of Cancer, Sun Yat-sen University Cancer Center, Guangzhou, 510060 Guangdong P. R. China

**Keywords:** Erythrogenesis, Platinum-containing antineoplastic drugs, Dormancy, Molecular mechanisms, Spontaneous tumor regression, Self-sampling technology, Non-infection-related cancer, Tumor angiogenesis, Epigenetic feature

## Abstract

To accelerate our endeavors to overcome cancer, *Chinese Journal of Cancer* has launched a program of publishing 150 most important questions in cancer research and clinical oncology. In this article, 10 more questions are presented as follows. Question 15: Can tumor-induced erythrogenesis provide qualified red blood cells for carrying oxygen to distant organs? Question 16: Can we overcome tumor resistance to platinum-containing antineoplastic drugs by activating the sensitivity factors in the tumor? Question 17: How can a cancer cell stay dormant for years? Question 18: Why do cancer cells use distinct transcriptomic and proteomic programs to reach the same metastatic phenotype? Question 19: Why do some cancers regress spontaneously? Question 20: What are the regulatory mechanisms occurring in donor cells that determine selective sorting of biological content into vesicles and their biological consequences in recipient cells? Are the genetic transfer and exchange of biological messages between cells transient? Is the phenotypic manipulation of recipient cells temporary or prolonged and persistent? If extracellular vesicles possess immune-modulatory potential, how could they be exploited for immune interventions and cancer immunotherapy? Presumably the cargo of extracellular vesicles reflects the cells of their origin and can be used for cancer diagnosis, how could the uniform/stringent capture criteria be met universally for applying EVs in point-of-care diagnostics for cancer patients? Question 21: Can we use self-sampling technologies to monitor the tumor genetic alterations for more precise targeted therapy? Can we cure a heterogeneous tumor by sequentially targeting the driver molecules? Question 22: Can we postpone the onset of non-infection-related cancers? Question 23: How many types of cells can jointly form the tumor vasculature to provide blood supply for tumor progression? Question 24: How tumor cells transmit their epigenetic features to daughter cells and maintain the malignant phenotype?

To accelerate our endeavors to overcome cancer, *Chinese Journal of Cancer* has launched a program of publishing 150 most important questions in cancer research and clinical oncology [1], with the first 14 questions been published in the last three issues [2–4]. In this article, Questions 15–24 are selected and presented. *Chinese Journal of Cancer* is still open to collect more key questions in cancer research and clinical oncology. Please send us your thoughtful questions to Ms. Ji Ruan via email: ruanji@sysucc.org.cn.

## Question 15: Can tumor-induced erythrogenesis provide qualified red blood cells for carrying oxygen to distant organs?

### Background and implications

It has been confirmed that some tumor cells can express erythropoietin and its receptor. Moreover, when we examine the tissue structures of clear cell renal cell carcinoma and hepatocellular carcinoma, numerous red blood cells in different maturation phases characterized by the cellular shapes could be observed. It is not surprising that the kidney and liver are initial organs for erythrogenesis during embryonic development. It is highly possible that solid tumors in these organs could generate red blood cells through the mechanism very different from that of typical erythrogenesis in the bone marrow. A very interesting question following the confirmation of tumor-induced erythrogenesis would be about how good those tumor-generated red blood cells are in terms of functioning as oxygen carriers. The exploration in this direction might be able to open a new field on using animal tumor models to robustly generate a large amount of red blood cells for clinical blood transfusion.

#### Submitter

Chao-Nan (Miles) Qian.

#### Affiliation

Sun Yat-sen University Cancer Center, Guangzhou, Guangdong, P. R. China.

qianchn@sysucc.org.cn.

## Question 16: Can we overcome tumor resistance to platinum-containing antineoplastic drugs by activating the sensitivity factors in the tumor?

### Background and implications

Platinum-containing antineoplastic drugs are chemotherapeutic agents to treat almost 50% of cancer patients. As the first member of this family, cisplatin has been used in combination chemotherapy for multiple tumor types. Initial therapeutic effects of platinum-containing antineoplastic drugs are usually high but cisplatin-resistant tumors will inevitably relapse, which happens in the majority of cancer patients. Many research teams have been exploring the resistant mechanisms for cisplatin treatment in the past several decades without clinically significant breakthrough.

We are thinking another way round: Could we enhance the therapeutic effects of platinum-based antineoplastic drugs by activating the sensitivity factors in the tumor? Obviously, the identification of sensitivity factors from sable cellular models is therefore critical to answer this question. The feasibility of this exploration has been preliminary verified. We have established a super-sensitive cellular clone to cisplatin treatment, S16, from its parental nasopharyngeal carcinoma cell line CNE2. Moreover, by using first-generation microarray technology, asparagine synthetase and matrix metalloproteinase 19 have been identified and functionally validated to be sensitivity factors for cisplatin treatment. How to activate the sensitivity mechanisms inside a solid tumor could be the main issue before clinical benefit could be obtained.

The implications of answering this question include enhancing the efficacy of platinum-containing antineoplastic drugs, prolonging the survival of cancer patients, and improving cure rate in cancer patients treated with platinum-based chemotherapy.

#### Submitter

Chao-Nan (Miles) Qian.

#### Affiliation

Sun Yat-sen University Cancer Center, Guangzhou, Guangdong, P. R. China.

qianchn@sysucc.org.cn.

## Question 17: How can a cancer cell stay dormant for years?

### Background and implications

Dormancy is very common in nature, in particular in the vegetable world where plant seeds can be dormant for years and only become to grow again after being planted.

This important biological property also allows a few neoplastic cells to survive in otherwise asymptomatic patients for years. The consequences are recurrence of the disease many years after the successful treatment of the primary tumor.

Dormancy has been proposed to be maintained by several different ways, e.g., by special cancer stem cells, immune surveillance, or a mitotic blockage; however, the full comprehension of its biology is still eluding us.

A better understanding of dormancy could lead to a quantum leap in controlling cancer.

#### Submitter

Francesco Pezzella.

#### Affiliation

Nuffield Division of Clinical Laboratory Science, University of Oxford, Oxford, UK.

francesco.pezzella@ndcls.ox.ac.uk.

## Question 18: Why do cancer cells use distinct transcriptomic and proteomic programs to reach the same metastatic phenotype?

### Background and implications

Transcriptomic and proteomic profiles of metastases versus primary tumors have been compared by several investigators. Profoundly distinct profiles have been revealed in most cases, with trends for similarity between pairs of metastases and primary tumors from individual patients. However, rare tight-matches were revealed even across different metastases originating from an individual tumor. Next-generation sequencing confirmed the complexity of such a scenario. These findings suggest distinct, albeit self-supporting, control modules for metastatic spreading.

Determining the underlying molecular mechanisms may be very helpful for a better understanding of the metastatic process. It may also help avoiding misleading classifications of metastatic factors. Ultimately, it may allow for a better design of therapeutic strategies for metastatic tumors.

#### Submitter

Saverio Alberti.

#### Affiliation

Unit of Cancer Pathology, Center of Excellence for Research on Aging & Translational Medicine, CeSI-MeT, “G. D’Annunzio” University Foundation, Chieti, Italy.

s.alberti@unich.it.

## Question 19: Why do some cancers regress spontaneously?

### Background and implications

Spontaneous tumor regression (STR) is one of the most fascinating phenomena observed in medical sciences. The first classic monograph on STR was laid by Tilden C. Everson and Warren H. Cole in 1956. Colorectal cancers, malignant melanomas, renal cell carcinomas, and some head and neck cancers are known to regress on their own. Various mechanisms have been postulated for STR, such as the induction of differentiation, apoptosis, tumor necrosis, and immunological, hormonal, psychological, and epigenetic mechanisms. The intra-tumoral immune reaction in the evolution of cancer is known; the hypothesis that cancer development is strictly controlled by host’s immune reaction has also been well established. The idea is to identify the underlying intra-tumoral response(s) in cancers which fully responded to standard treatment(s), without long-term recurrence, and compare the intra-tumoral responses in residual disease and recurrences. This research finding will open a new vista in cancer research.

#### Submitter

Manigreeva Krishnatreya.

#### Affiliation

Dr. Bhubaneswar Borooah Cancer Institute, Guwahati, India.

Email: manigreeva@gmail.com; manigreeva@bbci.in.

## Question 20: Question (1): What are the regulatory mechanisms occurring in donor cells that determine selective sorting of biological content into vesicles and their biological consequences in recipient cells? Question (2): Are the genetic transfer and exchange of biological messages between cells transient? Is the phenotypic manipulation of recipient cells temporary or prolonged and persistent? Question (3): If extracellular vesicles possess immune-modulatory potential, how could they be exploited for immune interventions and cancer immunotherapy? Question (4): Presumably the cargo of extracellular vesicles reflects the cells of their origin and can be used for cancer diagnosis, how could the uniform/stringent capture criteria be met universally for applying EVs in point-of-care diagnostics for cancer patients?

### Background and implications

Extracellular vesicles (EVs) are nano-sized vesicles (exosomes and microvesicles) secreted by almost every cell type under both normal and pathologic conditions. EVs contain a variety of proteins such as transcription factors as well as nucleic acids such as DNA and non-coding regulatory RNAs (1, 2). Exosomes are originated from endocytic compartments (3), relevantly reflecting the cytoplasmic content of their parent cells; microvesicles are shed directly from plasma membrane, comparatively reflecting the biology of cell surfaceome. It is important to consider that the molecular content and the secreted levels of EVs are differentially reflected in each cancer type and grade, making EVs a potential source of cancer biomarkers (3, 4).

Extracellular vesicles act as conveners of intercellular communication by exchanging biological information between cells (5–7) and are largely implicated in determining cell fates (8). Based on their inherent capability of transferring regulatory elements, EVs may elicit a newly evolved mechanism of trans-regulation between cells and confer genomic instability in recipient cells (1). The oncogenic content of EVs transferred from cancer cells to normal recipient cells could consequently induce malignant phenotypes (9–11). In a similar way, EVs could mediate an intercellular transfer of phosphatase and tensin homolog (PTEN)-targeting microRNAs to primary tumor cells in order to bypass tumor suppressor checkpoint and enable primary tumor cells to metastasize (12). As such, the EV-mediated dissemination of bioactive content contributes to cell phenotypic transitions, immune modulation, and re-shaping of tumor microenvironment (13). The most profound input of cancer-associated EVs is their participation in pre-metastatic niche formation by enabling cells to mobilize at new regions (14, 15). As such, EVs from various tumor types forecast organ-specific metastasis (organotropism) by preferentially fusing at their predicted destinations in target organs through EV-linked distinct integrins (16). Thus, EVs promote tumor organotropic metastasis and prepare favorable pre-metastatic niche for future metastasis.

Since cancer needs successful co-option with extracellular environment, tumor-derived EVs can condition tumor microenvironment for successful co-option of cancer cells in a given niche (12). For this, EVs transport pro-angiogenic growth factors that cope with nutrient requirements in microenvironment and favor the formation of blood vessels (17), or may undergo vessel co-option. It is tempting to escalate that tumor cells have evolved yet another mean of co-option through suppressing anti-tumor immune cells by secreting EVs loaded with immune-suppressing ligands (18). In fact, EVs carrying such ligands interact with corresponding receptors presented on immune cells and induce suppression or apoptosis of T cells and normal killer cells. This mechanism facilitates cancer cells to co-exist in tumor microenvironment by suppressing anti-tumor immune cells (18). Thus, given all observations together, EVs facilitate stochastic patterns of cancer physiology.

Despite multifaceted roles of EVs in cancer biology, some important questions about their regulatory mechanisms and their clinical applications are yet to be answered. On the biotech front, the answers to the above questions will improve the way of designing more precise therapeutic strategy for cancer patients. Collective efforts from academia and biotech industries are expected to translate EVs into a platform of innovative and personalized therapies.

### Acknowledgements

I acknowledge FAPESP (Sao Paulo Research Foundation, Proc. No. 12/24574–3), and CAPES (Coordination for the Improvement of Higher Education Personnel, Proc. No. BEX 7057/15-6)—Brazil.

**References**

1. Fatima F, Nawaz M. Vesiculated long non-coding RNAs: offshore packages deciphering trans-regulation between cells, cancer progression and resistance to therapies. Non-Coding RNA. 2017;3(1):10.
2. Mateescu B, Kowal E, Balkom B, Bartel S, Bhattacharyya S, Buzas E, et al. Obstacles and opportunities in the functional analysis of extracellular vesicle RNA. J Extracell Vesicles. 2017;6(2).
3. Nawaz M, Camussi G, Valadi H, Nazarenko I, Ekstrom K, Wang X, et al. The emerging role of extracellular vesicles as biomarkers for urogenital cancers. Nat Rev Urol. 2014;11(12):688–701.
4. Rak J. Extracellular vesicles—biomarkers and effectors of the cellular interactome in cancer. Front Pharmacol. 2013;4:21.
5. Turturici G, Tinnirello R, Sconzo G, Geraci F. Extracellular membrane vesicles as a mechanism of cell-to-cell communication: advantages and disadvantages. Am J Physiol Cell Physiol. 2014;306(7):C621–33.
6. Mathivanan S, Ji H, Simpson RJ. Exosomes: extracellular organelles important in intercellular communication. J Proteom. 2010;73(10):1907–20.
7. Ratajczak J, Wysoczynski M, Hayek F, Janowska-Wieczorek A, Ratajczak MZ. Membrane-derived microvesicles: important and underappreciated mediators of cell-to-cell communication. Leukemia. 2006;20(9):1487–95.
8. Nawaz M, Fatima F, Vallabhaneni KC, Penfornis P, Valadi H, Ekstrom K, et al. Extracellular vesicles: evolving factors in stem cell biology. Stem Cells Int. 2016;2016:1073140.
9. Al-Nedawi K, Meehan B, Micallef J, Lhotak V, May L, Guha A, et al. Intercellular transfer of the oncogenic receptor EGFRvIII by microvesicles derived from tumour cells. Nat Cell Biol. 2008;10(5):619–24.
10. Al-Nedawi K, Meehan B, Kerbel RS, Allison AC, Rak J. Endothelial expression of autocrine VEGF upon the uptake of tumor-derived microvesicles containing oncogenic EGFR. Proc Natl Acad Sci U S A. 2009;106(10):3794-9.
11. Skog J, Wurdinger T, van Rijn S, Meijer DH, Gainche L, Sena-Esteves M, et al. Glioblastoma microvesicles transport RNA and proteins that promote tumour growth and provide diagnostic biomarkers. Nat Cell Biol. 2008;10(12):1470-6.
12. Zhang L, Zhang S, Yao J, Lowery FJ, Zhang Q, Huang WC, et al. Microenvironment-induced PTEN loss by exosomal microRNA primes brain metastasis outgrowth. Nature. 2015;527(7576):100-4.
13. Fatima F, Nawaz M. Stem cell-derived exosomes: roles in stromal remodeling, tumor progression, and cancer immunotherapy. Chin J Cancer. 2015;34(12):541–53.
14. Peinado H, Aleckovic M, Lavotshkin S, Matei I, Costa-Silva B, Moreno-Bueno G, et al. Melanoma exosomes educate bone marrow progenitor cells toward a pro-metastatic phenotype through MET. Nat Med. 2012;18(6):883–91.
15. Costa-Silva B, Aiello NM, Ocean AJ, Singh S, Zhang H, Thakur BK, et al. Pancreatic cancer exosomes initiate pre-metastatic niche formation in the liver. Nat Cell Biol. 2015;17(6):816–26.
16. Hoshino A, Costa-Silva B, Shen TL, Rodrigues G, Hashimoto A, Tesic Mark M, et al. Tumour exosome integrins determine organotropic metastasis. Nature. 2015;527(7578):329–35.
17. Fong MY, Zhou W, Liu L, Alontaga AY, Chandra M, Ashby J, et al. Breast-cancer-secreted miR-122 reprograms glucose metabolism in premetastatic niche to promote metastasis. Nat Cell Biol. 2015;17(2):183–94.
18. Nawaz M, Fatima F, Nazarenko I, Ekstrom K, Murtaza I, Anees M, et al. Extracellular vesicles in ovarian cancer: applications to tumor biology, immunotherapy and biomarker discovery. Expert Rev Proteom. 2016;13(4):395–409.

#### Submitter

Muhammad Nawaz.

#### Affiliation and email

Department of Pathology and Forensic Medicine, Ribeirao Preto Medical School, University of Sao Paulo, Ribeirao Preto, Brazil.

nawazm.edu@gmail.com.

## Question 21: Question (1): Can we use self-sampling technologies to monitor the tumor genetic alterations for more precise targeted therapy? Question (2): Can we cure a heterogeneous tumor by sequentially targeting the driver molecules?

### Background and implications

Solid tumors are heterogeneous in nature, and each portion of the tumor might rely on different signaling pathways for growth and spread. Moreover, tumor cells have a capacity to develop resistance to a single molecule-targeted agent via the up-regulation of the partially inhabited pathway, mutation of the target gene, or activation of alternative pathway. However, it is hypothesized that driver molecules for tumor cell survival and spread are timely and spaciously existing. Clearly, a dynamic monitoring on tumor genetic alterations to indentify the driver molecules is needed to test this hypothesis and to improve therapeutic design. For practicable reason, self-sampling technologies can facilitate the dynamic monitoring in an economic way. The biological samples for self-sampling could be any types of body fluid or a drop of blood which are easy to collect and preserve. Answering the above-mentioned questions will further improve our precision medical practice and hopefully cure more cancer patients.

#### Submitter

Mei Wang.

#### Affiliation

Department of Opthhalmology, Sun Yat-sen Memorial Hospital, Sun Yat-sen University, Guangzhou, Guangdong, P. R. China.

13724856155@163.com.

## Question 22: Can we postpone the onset of non-infection-related cancers?

### Background and implications

The formation of approximately two-thirds of cancers in all types has no close relationship with infection. The etiology of these cancers is mostly undetermined, while a significant portion of them is thought to be resulted from genetic susceptibility. Two perfect examples of genetic susceptibility are adenomatosis polyposis coli (APC)-mutated colorectal cancer and breast cancer early onset 1/2 (BRCA1/2)-mutated breast cancer. Although the genetic susceptibility exists on day one of birth, the cancers usually occur in the mid-life of the patients, giving the hosts several decades of cancer-free survival. In 2010, I hypothesized that there should be a postponing mechanism in the host to delay the cancer onset. I named the key molecules for delaying cancer onset as “postponers.” If we could amplify the to-be-validated postponing mechanism, we probably can postpone the cancer onset to a late-life period of the patients, providing them several more decades of cancer-free survival. Moreover, if the postponing efforts are more profound, the hosts might not die of cancer in their whole life, avoiding those prophylactic yet destructive medical approaches to remove the high-risk organs from the host.

#### Submitter

Chao-Nan (Miles) Qian.

#### Affiliation

Sun Yat-sen University Cancer Center, Guangzhou, Guangdong, P. R. China.

qianchn@sysucc.org.cn.

## Question 23: How many types of cells can jointly form the tumor vasculature to provide blood supply for tumor progression?

### Background and implications

The growth and spread of a solid tumor usually depend on the establishment of effective vasculature system inside and adjacent to the tumor. A classical concept is that tumor angiogenesis is resulted from the proliferation and migration of peripheral differentiated endothelial cells, with spouting angiogenesis to be the most frequently studied pattern of tumor angiogenesis. Another important source for forming tumor vasculature is cycling endothelial progenitor cells from the bone marrow, and the lining of endothelial progenitor cells that forms blood conduits is named vasculogenesis. Recently, the concept of vessel co-option, hijacking the blood vessels in the normal tissue surrounding the tumor, has been recognized to be critical in forming the vasculature of primary tumor and secondary metastatic tumor. The process of vessel co-option is usually accompanied by remodeling of the vessels adjacent to the tumor prior to being co-opted, as well as the co-opted vessels inside the solid tumor. In melanoma, glioblastoma, and other solid tumors, vascular mimicry in the tumor, which is formed directly by the lining of cancer cells, has been found for years. Obviously, different manners of forming blood vessels commonly exist at the same time to support tumor progression.

We have previously found that at least three types of blood vessels exist in clear cell renal cell carcinoma, namely, undifferentiated micro-vessels (expressing CD31 but not CD34 protein in the cells forming micro-vasculature, without pericyte support), immature differentiated micro-vessels (expressing both CD31 and CD34 proteins on the cells, but not supported by pericytes), and mature differentiated vessels (expressing both CD31 and CD34 proteins, with supporting pericytes partially cover the outer layer of the vessels). The mature differentiated vessels are usually co-opted vessels in clear cell renal cell carcinoma. However, the origins of the undifferentiated as well as the immature differentiated vessels remain unclear.

An interesting question is therefore raised: How many types of cells can join the formation of effective blood conduit for tumor progression? Answering this question will pave the road for developing more effective antiangiogenic therapeutics.

#### Submitter

Chao-Nan (Miles) Qian.

#### Affiliation

Sun Yat-sen University Cancer Center, Guangzhou, Guangdong, P. R. China.

qianchn@sysucc.org.cn.

## Question 24: How tumor cells transmit their epigenetic features to daughter cells and maintain the malignant phenotype?

### Background and implications

It has become increasingly evident that the development of tumor involves both genetic and epigenetic changes. The term of “epigenetics” refers to machineries that alter gene expression without affecting the primary DNA sequence, including DNA methylation, histone modifications, and non-coding RNA expression. Epigenetic features to some extent associate with the expression pattern of genes that drive cell division and differentiation. Altered epigenetic modifications, such as CpG island hypermethylation-induced silencing of tumor suppressors, contribute to the malignant phenotype of tumor cells. The epigenetic features on chromatin play a central role in maintaining transcriptional patterns during tumorgenesis. How daughter cells “inherent” epigenetic modifications precisely from their predecessor during cell division, however, remains largely unknown.

We and Denny Reinberg’s group provided proof of a principle that cyclin-dependent kinase 1/2 (CDK1/2) phosphorylates enhancer of zeste homolog 2 (EZH2), a core component of polycomb repressive complex 2 (PRC2) catalyzing H3K27me3, at Thr350/345 site during cell cycle which facilitates the recruitment of PRC2 to target locus in a long non-coding RNA-dependent manner and establishes H3K27me3 on newly synthesized chromatin (1, 2). More answers for this question would benefit for the development of new therapeutics that possibly corrects the altered epigenetic machinery for cancer treatment.

**References**

1. Chen S, Bohrer LR, Rai AN, Pan Y, Gan L, Zhou X, Bagchi A, Simon JA, Huang H.Cyclin-dependent kinases regulate epigenetic gene silencing through phosphorylation of EZH2. Nat Cell Biol. 2010;12(11):1108–14.
2. Kaneko S, Li G, Son J, Xu CF, Margueron R, Neubert TA, Reinberg D. Phosphorylation of the PRC2 component Ezh2 is cell cycle-regulated and up-regulates its binding to ncRNA. Genes Dev. 2010;24(23):2615–20.

#### Submitter

Shuai Chen.

#### Affiliation and email

Sun Yat-sen University Cancer Center, Guangzhou, Guangdong, P. R. China.

chenshuai@sysucc.org.cn.

**References**

1. Qian CN, Zhang W, Xu RH. Defeating cancer: the 150 most important questions in cancer research and clinical oncology. Chin J Cancer. 2016;35:104.
2. Wee JT, Poh SS. The most important questions in cancer research and clinical oncology: question 1. Could the vertical transmission of human papilloma virus (HPV) infection account for the cause, characteristics, and epidemiology of HPV-positive oropharyngeal carcinoma, non-smoking East Asian female lung adenocarcinoma, and/or East Asian triple-negative breast carcinoma? Chin J Cancer. 2017;36:13.
3. Venniyoor A. The most important questions in cancer research and clinical oncology: question 2–5. Obesity-related cancers: more questions than answers. Chin J Cancer. 2017;36:18.
4. Chinese Journal of Cancer. The most important questions in cancer research and clinical oncology: question 6–14. Chin J Cancer. 2017;36:33.

